# Relationship of Parieto-Occipital Brain Energy Phosphate Metabolism and Cognition Using ^31^P MRS at 7-Tesla in Amnestic Mild Cognitive Impairment

**DOI:** 10.3389/fnagi.2020.00222

**Published:** 2020-08-28

**Authors:** Namrata Das, Jimin Ren, Jeffrey S. Spence, Audette Rackley, Sandra B. Chapman

**Affiliations:** ^1^Center for BrainHealth, The University of Texas at Dallas, Dallas, TX, United States; ^2^Advanced Imaging Research Center, and Department of Radiology, University of Texas Southwestern Medical Center, Dallas, TX, United States

**Keywords:** brain energy metabolism, membrane phospholipid, amnestic mild cognitive impairment (aMCI), Alzheimer’s disease (AD), adenosine triphosphate (ATP), ^31^phosphorus magnetic resonance spectroscopy, ^18^fluorodeoxyglucose positron emission tomography (^18^FDG PET)

## Abstract

**Background:**

The human brain has high energy requirements that continuously support healthy neuronal activity and cognition. A disruption in brain energy metabolism (BEM) may contribute to early neuropathological changes such as accumulation of β-amyloid and tau in vulnerable populations. One such population is amnestic mild cognitive impairment (aMCI) where some individuals are at risk for developing dementia, i.e. Alzheimer’s disease (AD). Recent advances in imaging technology are providing new avenues to measure BEM accurately using 31phosphorus magnetic resonance spectroscopy (31P MRS) at ultra-high-field (UHF) magnetic strength 7-Tesla. This study investigates whether a methodology using partial volume-coil 31P MRS at 7T over parieto-occipital lobes can accurately quantify high-energy phosphate and membrane phospholipid metabolites in aMCI. A secondary objective was to explore BEM and membrane phospholipid indices’ correspondence with cognitive performance in domains of executive function (EF), memory, attention, and visuospatial skills in aMCI, a heterogeneous population.

**Methods:**

19 aMCI participants enrolled in the study completed cognitive assessment and 31P MRS scan. BEM indices were measured using three energy indicators: energy reserve (PCr/t-ATP), energy consumption (intracellular_Pi/t-ATP), and metabolic state (PCr/intracellular_Pi) along with regulatory co-factors of BEM-intracellular Mg^2 + ^ and pH; whereas the ratio of phosphomonoesters (PMEs) to phosphodiesters (PDEs) – membrane phospholipid indicator.

**Results:**

31P MRS scan showed thirteen well-resolved peaks with precise quantification of the phosphorus metabolites at UHF. The higher BEM indices were associated with lower cognitive performance of memory [(energy reserve indicator: CVLT *p* = 0.004), (metabolic state indicator: CVLT *p* = 0.007)], executive function [(metabolic state indicator: TOSL (*p* = 0.044)], and attention [(pH: selective auditory task, *p* = 0.044)]. The finding of an inverse relationship observed in the parieto-occipital lobes suggests an association between neuronal energy markers with cognition in aMCI.

**Conclusion:**

The significant contribution of this preliminary research was to establish the feasibility of utilizing a methodology at UHF to accurately measure high-energy phosphate and membrane phospholipid metabolites in a population with heterogeneous outcomes. This work offers a novel approach for future work to further elucidate early dementia biomarkers or precursors to the downstream accumulation of amyloid and tau using the combination of MRS-PET imaging modalities in AD.

## Introduction

Mild cognitive impairment (MCI) represents a heterogeneous clinical condition that is definable and objectively classifiable; however, its nature is unstable. MCI appears to follow one of three courses: (1) static condition with little to no change; (2) revert back to normal cognitive function; and (3) advance to dementia, especially Alzheimer’s disease (AD) (Albert et al., 2011; Petersen et al., 2014). Motivated by the very nature of this heterogeneity, MCI may present with an ideal group to examine whether the relationship between cognitive and brain energy metabolism (BEM) exists. Such preliminary efforts may help elucidate some possible predictors that could subsequently be investigated in longitudinal studies and compare with cognitively normal and AD population in a pilot trial testing a new methodology at ultra-high-field (UHF) MRI.

Traditionally in MCI, cognitive decline is presented as single or multiple-domain deficits in which either one or more cognitive function decline is observed (Alichniewicz et al., 2012; Cerami et al., 2014; Giau et al., 2019). A subset of the MCI population is classified as amnestic MCI (aMCI). This aMCI subgroup manifests significant memory problems with or without alterations in other cognitive domains for their age, but they remain functionally independent (Petersen’s et al., 2001). An estimated 30% of MCI subjects are of the aMCI-type with this type showing a higher predilection to subsequently evolve into criteria to meet diagnosis of AD (Cerami et al., 2014). The conversion rate of AD/dementia diagnosis is much higher in the general category of MCI, ranging from 10–15% per year in the clinical setting and 6–10% in population-based studies when compared to age-matched healthy comparison group. In the latter group, the conversion rate is estimated to be about 1–2% per year (Petersen et al., 1999; Farias et al., 2009; Shimada et al., 2019). A meta-analysis in 25 studies supported an overall reversion rate from MCI back to cognitively normal as approximately 24% (Malek-Ahmadi, 2016). Given the heterogeneity of MCI, it is important to point out that although this group may be at greater risk of developing dementia than the normal population, a significant proportion of this population still does not show AD’s frank symptoms to reach diagnostic criteria. Overall, this varying range of MCI progression to dementia, or reversal back to normal, supports a plausible hypothesis that the biological mechanisms involved are heterogeneous and unstable. As such, this classifiable group may provide a valuable population to explore the early biomarkers of neuronal function that affect cognition before the diagnosis of dementia.

Due to the heterogeneity, unstable nature, and wide range of cognitive behavior in individuals with MCI, research is focused on further elucidating novel biomarkers occurring inside the brain cells in this vulnerable complex group. These novel biomarkers are speculated to contribute to our knowledge about possible early pathomechanistic disruptions that could later inform which individuals go on to develop AD and who do not. Previous work established that extracellular deposits of β-amyloid peptides and intracellular deposits of tau aggregates are associated with the pathology of MCI and AD (Mufson et al., 2012). Moreover, only the burden of intracellular tau is strongly correlated with the degree of cognitive decline in MCI and AD (Bejanin et al., 2017). The correlation between tau and cognition observed in postmortem human brain is replicated in *in vivo* studies using a positron emission tomography (PET) tau-radiotracer ^18^F-AV-1451 scan (Bejanin et al., 2017). However, little is known about the initial process that disrupts the internal biological mechanisms causing tau hyperphosphorylation leading to the aggregation of tau in the form of neurofibrillary tangles and the relationship of these early biological markers with cognition in MCI.

Growing evidence in MCI and AD pathology supports a role of neuroenergetics, a study of BEM along with depletion of neurotransmitters and neuroinflammation as significant contributing factors to the early disease pathophysiology and progression even before the accumulation of β-amyloid and tau (Ogawa et al., 1996; Heneka et al., 2015). It is intriguing to know that the human brain weighs only 2% of the body weight, whereas it consumes about 25% of the glucose and 20% of the body’s oxygen to produce energy in adenosine triphosphate (ATP) (Zlokovic, 2011). The energy produced supports various cellular events such as Na^+^/K^+^-ATP activities to maintain membrane potential, integrity, and metabolite exchange for neuronal firings (Albers and Siegel, 1999). In sum, the high-energy requirements of the brain make it a highly vulnerable organ to BEM disturbances, especially in the transitory stage such as MCI. It has been speculated that disruptions in BEM and neurochemicals may represent early disease markers in MCI who may convert to AD/dementia with prominent cognitive decline and is observed versus non-converters.

The hypothesis of BEM alterations in MCI can be supported by two recent studies using ^18^FDG-PET to measure brain glucose metabolism. Earlier longitudinal study of 3 years in 45 MCI (37 aMCI and 8 non-amnestic) using 18-FDG PET showed a distinct pattern of neurometabolic changes in terms of reduced glucose metabolism in the temporoparietal brain areas in individuals associated with cognitive deterioration who progressed toward AD (Cerami et al., 2014). In the 3-year follow-up, 14 MCI (11 aMCI and 3 non-amnestic MCI) with normal glucose metabolism in the brain never converted to dementia, whereas 24 MCI of the 45 enrolled (18 aMCI and 6 non-amnestic MCI) with region-specific reduced glucose metabolism in the brain showed increasing cognitive decline to the point where they converted to AD/other dementias (Cerami et al., 2014). Similarly, in a different retrospective longitudinal study of 4 years in 30 aMCI using ^18^FDG PET, patients without typical hypometabolism in temporoparietal areas did not convert to AD, and cognitive function remained stable over the period of follow-up (Cerami et al., 2018). Thereby, emphasizing that brain metabolism plays a crucial role in regulating cognitive behavior, paving the path to explore the relationship of BEM-cognitive correlates in aMCI.

In addition to ^18^FDG-PET, magnetic resonance spectroscopy (MRS) represents an emerging brain imaging field that shows promise in providing a platform to investigate BEM, neuroinflammatory, and excitotoxicity markers at the microscopic level inside the brain cells. These microscopic changes have the potential to later affect the macroscopic neural structure, such as the brain’s anatomy and physiology, subsequently leading to brain atrophy, which are well studied in MCI and AD (Godlewska et al., 2017). Specifically, the high-energy phosphate metabolites a methodology to investigate BEM at the molecular level is investigated by using a novel non-invasive imaging technology—phosphorus (^31^P) MRS. This technology aids in the quantification of high-energy phosphate metabolites [ATP, phosphocreatine (PCr), and inorganic phosphate (Pi)] and membrane phospholipid metabolites [phosphoethanolamine (PE), phosphocholine (PC), glycerophosphoethanolamine (GPE), and glycerophosphocholine (GPC)] (Ross and Sachdev, 2004) crucial to understand BEM and membrane phospholipid metabolism. Recent work by Rijpma et al. (2018) using whole-brain ^31^P MRS at 3Tesla observed that the Pi/PCr ratio and the membrane phospholipid metabolites, especially PE, GPC, and GPE, were lower in mild AD compared to age-matched healthy controls. One possible explanation of decreased Pi/PCr findings in AD may be due to a reduction of creatine kinase (CK) activity, an enzyme of mitochondria, thereby increasing PCr (David et al., 1998; Rijpma et al., 2018). Moreover, a ^31^P MRS study at 1.5T showed an association between higher levels of PE and PC in the prefrontal cortex with the severity of cognitive decline measured using the Cambridge Cognitive scale, which included domains of memory, orientation, language, attention, praxis, calculation, abstract thinking, and visual perception subtests (Forlenza et al., 2005). Similarly, other studies in AD have identified higher levels of PE and PC membrane phospholipid metabolites either in temporoparietal areas (Brown et al., 1989) or over diffuse cortical areas (Pettegrew et al., 1988). ^31^P MRS methodology may aid in fine-tuning the characterization of MCI group who will progress to dementia or not in addition to hallmark proteinopathies-accumulation of amyloid and tau.

Over the last two decades, the ^31^ P MRS technique has been used in brain imaging at lower magnetic strength. Nonetheless, we have at least two main unanswered questions in MCI: (1) Can BEM metabolites be measured in the early stage with higher resolution compared to the existing work using lower magnetic strength and, (2) Is there a correspondence between BEM and cognitive performance in this group? The main challenge is to quantify the high-phosphate energy metabolites accurately and precisely. At lower magnetic strength, like 3T or less, neurochemicals or neurometabolites with only high signal-to-noise ratio (SNR) and abundant in nature can be assessed (Oeltzschner et al., 2019). However, recent advancements in MRI technology to use UHF magnets have enabled the robust application of the ^31^P MRS technique (Godlewska et al., 2017). UHF increases the SNR with a higher spectral resolution with enhanced sensitivity of neurochemicals and neurometabolites, which are present in minute concentrations (Mekle et al., 2009; Pradhan et al., 2015; Oeltzschner et al., 2019). To date, no study has used the UHF methodology of ^31^P MRS in understanding the relationship of high-energy phosphate and membrane phospholipid metabolites relationship with cognition in an aMCI group.

The present pilot study in aMCI by 7T ^31^P MRS had two goals. The first goal was to conduct a preliminary study to determine the feasibility of quantifying various high-energy phosphate and membrane phosphorus metabolites identifiable at 7-Tesla using a partial volume-coil in the parieto-occipital lobes of an aMCI cohort. The current study represents the first known project to test whether a methodology, previously shown to separate similar metabolites in the healthy young adult brain (Ren et al., 2015), heart (Rodgers et al., 2014), and muscles (Ren et al., 2013) could be adapted to measure BEM using ^31^P MRS at 7 Tesla in participants classified as aMCI. We selected parieto-occipital lobes as the region of interest based on two previous MCI studies (Ashraf et al., 2015; Corriveau-Lecavalier et al., 2019). Ashraf’s et al. (2015) research supported increased metabolism (hypermetabolism) in the occipital cortex in MCI compared to healthy controls using ^18^FDG PET. Similarly, a longitudinal study in 26 MCI by Corriveau-Lecavalier et al. (2019) found that parietal lobe hyperactivation in 13 MCI was an early indicator in individuals who progressed to dementia. In MCI, based on Ashraf’s and Corriveau’s findings in the parietal and occipital lobes separately, we hypothesized that there may be compensatory hyperactivation, which may be a proxy of higher cerebral blood flow and energy metabolism during the early phases of biological changes, which may be associated with worsening of cognitive performance.

Our next goal was to investigate the relationships across *BEM indices* measured using the metabolite ratios of PCr/t-ATP (reflective of energy reserve), (Pi/t-ATP (reflective of energy consumption), and PCr/Pi (reflective of a metabolic state) and its regulatory co-factors (intracellular Mg^2+^ and pH) followed by *membrane phospholipid metabolite index* (PMEs/PDEs) in the parieto-occipital lobes with the cognitive performance of executive function (EF) (complex abstraction, innovation, inhibition and switching, conceptual reasoning, and working memory), memory (episodic), attention, and visuospatial skills. In this research, we define the energy reserve indicator the ratio of PCr to ATP, where PCr is the immediate energy reserve replenishing consumed ATP for maintaining ATP homeostasis during the neuronal activity. In contrast, Pi to t-ATP ratio reflects the relationship between metabolic substrate and product and for energy consumption (ATP → Pi + ADP) and reproduction (Pi + ADP → ATP). Pi tends to accumulate when cellular ATP consumption temporarily exceeds the ATP production by mitochondria at a steady state, and increased Pi in turn tends to increase the potential of energy production. The ratio of PCr to Pi is a metabolic state indicator because both PCr and Pi increase the turnover rate or production of ATP. This index was previously termed the oxidative phosphorylation index to measure mitochondrial function (Chance et al., 1981). Based on Ashraf’s and Corriveau’s studies in MCI, we hypothesized that higher BEM and membrane phospholipid metabolite indices in parieto-occipital lobes would be associated with lower performance on cognitive measures. Overall, in aMCI, which is considered a heterogeneous clinical condition, this work represents a preliminary effort to test a methodology at UHF to measure BEM metabolites precisely followed by exploring the BEM-cognitive performance correlates.

## Materials and Methods

### Protocol Approvals and Consent

The study was approved to include individuals with memory complaints between the age of 50 and 80 years by the Institutional Review Board of The University of Texas Southwestern Medical Center and The University of Texas at Dallas. Informed consent and HIPPA forms were signed as per the ethical standards of the Committee on Human Experimentation under Declaration of Helsinki, 1981.

### Participants

Nineteen aMCI participants were recruited using a multi-stage phone screen, including questions related to demographics and medical history along with a review of medications list, an imaging screen, and a memory screen called Clinical Dementia Rating (CDR) scale. Individuals with a history of metal in the body, neurological diseases, substance abuse, psychiatric problems, or any medication that stimulated or slowed the brain’s electrical activity were excluded. Eligible participants included for the cognitive screen and assessment were selected irrespective of gender and ethnic factors. All selected participants were right-handed and native English speakers with a minimum of 12 years of education and CDR scale score of 0.5, that is, subjective memory complainers.

#### Characterization of aMCI Participants

The qualified individuals on the phone screen with subjective memory complains (CDR of 0.5) were diagnosed as aMCI based on the established objective memory decline as per either Petersen’s or the Alzheimer’s disease Neuroimaging Initiative (ADNI) criteria, respectively. The comprehensive ADNI aMCI criteria implemented were: (1) subjective memory complaints; (2) CDR 0.5; (3) objective memory loss measured by logical memory subtest (Wechsler Memory Scale-III); (4) normal daily living activities and cognitive functions assessed using Lawton instrumental activities of daily living scale; (5) clinical dementia rating scale score of 0.5; (6) Mini-Mental Status Examination (MMSE) of 24–30; and (7) absence of dementia. In the Petersen’s criteria, all the characteristics mentioned above in ADNI were the same except objective memory loss was assessed using California Verbal Learning Task (CVLT) and individuals who scored −1.5 standard deviation (SD) below the age and education-adjusted mean were included in the study as aMCI. Cognitive screens such as logical memory and CVLT along with geriatric depression scale (short form) were included during the 3-h cognitive assessment protocol. Only participants with no or mild depression were included in the study. Table 1 summarizes all the screening measures and questionnaires administered to eligible aMCI participants.

**TABLE 1:** Screening measures and questionnaires in amnestic mild cognitive impairment (aMCI).

	Measures	Description
Screening Measures	Clinical Dementia Rating Scale (CDR) (Morris, 1993) Logical Memory (ADNI Criteria, WMS-III, Wechsler, 1997b) California Verbal Learning Task (Petersen’s et al., 2001) Mini Mental Status Examination (Folstein et al., 1975) Activities of daily living: Lawton Instrumental Activities of Daily Living Scale (Lawton et al., 2003)	Assesses six domains of cognitive and functional performance in memory, judgment and problem solving, community affairs, orientation, personal care and hobbies. Score: 0.5 The ability to recall a short story as it is read out immediately and after 25 min interval was assessed. Score: Delayed memory recall of 9–11 for 16 years of education or 5–9 for 8–15 years of education. The ability to recall a list of sixteen (16) words in four categories immediately after the list was read followed by delayed recall after 20 min interval was assessed. Score: −1.5 standard deviation below the mean for age and sex adjusted scores. Assesses the ability to examine functions of registration, attention and calculation, recall and language. Score: 24–30 Assesses the activities of daily living in the areas of ability to use phone, shopping, food preparation, housekeeping, laundry, mode of transportation, responsibility for own medications, and ability to handle finances. Score: 8
**Questionnaires** Subjective memory perception Depression	Multifactorial Memory Questions (MMQ) (Troyer and Rich, 2002) Geriatric Depression scale (Yesavage et al., 1982)	Assessed individual’s self-perception of memory in three subscales using 57 items questionnaire 1. MMQ-Contentment (MMQ-C): Self-satisfaction of memory 2. MMQ-Ability (MMQ-A): Self-perception of memory 3. MMQ-Strategy (MMQ-S): Using of memory strategies in daily life functions. Assessed the depression of the individuals using 15 items questionnaire. Score: **≤**6

### Cognitive Screen and Assessment

The cognitive screening measures included were MMSE, logical memory, and CVLT along with geriatric depression scale (Table 1). The other measures included in the present analyses were the cognitive domains of EF (complex abstraction, innovation, switching and inhibition, reasoning, and working memory), memory (episodic memory), attention (selective attention), and visuo-spatial skills. After the cognitive screening, individuals who met the criteria of aMCI and completed other cognitive assessments were invited to complete a 45-min ^31^P MRS scan at 7-Tesla. Table 3 summarizes all the neurocognitive measures implemented in this part of the study. The demographics/characteristics of the participants are presented in Table 4.

**TABLE 2:** Independent variables: energy and membrane phospholipid metabolite indices in amnestic mild cognitive impairment.

Index	Ratios	Definition
**BEM indices**		
Energy reserve indicator	PCr/t-ATP	Energy reserve indicator is the ratio of PCr/t-ATP as PCr is the immediate energy reserve metabolite replenishing energy demands during the neuronal activity
Energy consumption indicator	Pi/t-ATP: Extracellular-Pi/t-ATP Intracellular-Pi/t-ATP	Energy consumption indicator is the ratio of Pi/t-ATP as Pi is rapidly consumed to release energy in the form of ATP to support ongoing metabolic and neuronal activity.
Metabolic state indicator	PCr/Pi PCr/Intracellular-Pi	Metabolic state indicator is the ratio of PCr/Pi as both PCr and Pi increase the turnover rate or production of ATP. Previously this index was termed as oxidative phosphorylation index to measure mitochondrial function (Chance et al., 1981).
Regulatory co-factors	Magnesium (Mg^2+^) Intracellular pH	
**Membrane phospholipid index**		
Membrane phospholipid index	PMEs/PDEs	Membrane phospholipid index is the ratio of PMEs (the major precursors of phospholipids contributing to membrane synthesis) to PDEs (major products of phospholipid breakdown) indicating neuronal and non-neuronal membrane integrity (Ross and Sachdev, 2004).

*BEM, brain energy metabolism; PCr, phosphocreatine; t-ATP, total adenosine triphosphate; Pi, inorganic phosphate- internal or external; Mg^2+^, magnesium; PMEs, phosphomonoesters; PDEs, phosphodiesters.*

**TABLE 3:** Dependent variables: neurocognitive battery administered to amnestic mild cognitive impaired participants.

Cognitive domain	Measures	Description
**Executive function** 1. Complex abstraction	Test of Strategic Learning (TOSL) (Chapman et al., 2002) WAIS-III similarities (Wechsler, 1997a)	Assesses the ability to condense and synthesize complex information written as summary from a complex text. Scores represents number of abstracted ideas. Assesses the ability to think abstractly and to find similarities among words or ideas that may not appear to be similar on the surface.
2. Innovation	Test of Strategic Learning (TOSL) (Chapman et al., 2002)	Assesses the ability to construct as many interpretations as possible from a lengthy text to measure idea fluency.
3. Inhibition and switching	Trails B (Delis et al., 2001)	Assesses the ability to alternate between a number and letter by drawing a continuous line to measure the speed of processing.
4. Conceptual reasoning 5. Working memory	Delis–Kaplan executive function system (DKEFS) card sort (Delis et al., 2001) Digit Span Backwards Test (WMS-III, Wechsler, 1997b)	Assesses the ability to draw similarities between two sets of cards by drawing reasons behind the selection of cards was assessed. Assesses the ability to repeat a series of numbers backward.
**Memory** Episodic memory	Memory for facts: Test of Strategic Learning (TOSL) (Chapman et al., 2002)	Assesses the ability to recall details of a lengthy text.
Attention	Selective Auditory Learning Task (Hanten et al., 2007) Digit Span Forward Task (WMS-III, Wechsler, 1997b)	Assesses the ability to focus and pay attention to high-priority stimulus, while simultaneously blocking or inhibiting unwanted or low-priority information was assessed. Assesses the ability to pay attention and remember a series of number in the same sequence.
Visuospatial skills	Trails A (Delis et al., 2001)	Assesses the ability to visually search for numbers in ascending order and draw a continuous line to measure the speed of processing.

**TABLE 4:** Characteristics of the amnestic mild cognitive impairment participants.

Demographics	Mean ± SD	Range
Total number of participants *(n)*	19	
Age (years)	63.73 ± 7.62	50–76
Gender (M/F)	5/14	
Education (years)	17.79 ± 3.34	12–29
MMSE	27.89 ± 1.41	27–30
Geriatric depression scale	2.10 ± 1.76	0–6

### ^31^Phosphorus Magnetic Resonance Spectroscopy (^31^P MRS) Data Acquisition Protocol

^31^P MRS was performed at 7-Tesla MR system (Philips Healthcare, Cleveland, OH, United States), using a double-tuned ^1^H/^31^P partial volume-coil consisting of two-tilted partially overlapping 10 cm loops with a plastic housing fit to the shape of the head posterior. All the aMCI participants were positioned head-first and supine in the MRI scanner to acquire spectral data from the parieto-occipital lobes. Prior to this MRS data acquisition, axial, coronal, and sagittal T2-weighted turbo spin echo MR images were recorded for planning the shimming voxel. Typical imaging parameters included field-of-view 180 × 180 mm, repetition time (TR) 2.5 s, echo time 80 ms, turbo factor 15, in-plane spatial resolution 0.6 × 0.7 mm^2^, slice thickness 8 mm, gap 2 mm, bandwidth 517 Hz, number of acquisitions one, and acquisition time 2.1 min. Second-order ^1^H-based automatic volume shimming was applied.

Quantitative ^31^P MR spectra were acquired using a non-localized block-shaped excitation pulse with B_1_ 59 μT, and pulse width 0.2 ms with an estimated flip angle 55°, which was previously calibrated. B1 calibration of partial volume-coil is presented in the Supplementary Figure S1. The transmitter frequency was centered at 700 Hz upfield from the resonance of PCr. A short delay time of 0.5 ms was applied prior to free induction decay (FID) data collection to filter out broad-membrane phospholipids signal. The sampling point was 4 k, zero-filled to 8 k prior to Fourier transformation. For quantitative comparison of different metabolite ^31^P peaks, fully relaxed spectra were recorded at long TR of 25 s with and eight scan average. For Mg^2+^ measurement, to minimize measurement error due to potential T_1_ effects and time-of-the-day-dependent Mg^2+^ fluctuation, additional ^31^P spectra were also acquired at short TRs ranging from 1 to 5 s with 32 scan averages for a duration of 30 min. The chemical shifts of all ^31^P metabolites were referenced to PCr at 0 ppm. Gaussian apodization (6 Hz) was applied to each FID prior to Fourier transformation using the scanner software (SpectroView, Philips Healthcare). Precautions were taken to avoid signal contribution from the neck muscles by positioning the head chin-up, aligning the parieto-occipital region of the brain at the coil iso-center, and placing a supporting cushion pad underneath the neck.

### ^31^P MRS Data Analysis

First, the preprocessing of the raw data, including zero filling, apodization, Fourier transformation and phase correction, was performed at the scanner using the software package SpectroView from Philips Healthcare. The data post-processing, including baseline correction and spectral fitting, was done with an in-house program written in MATLAB. The fitting was based on the Voigt lineshape model (a combination of Gaussian and Lorentzian lineshape). Thirteen resonance phosphorus peaks were fitted: PCr, ATP (α-, β-, and γ-spins) nicotinamide adenine dinucleotide (total NAD), uridine diphosphate glucose (UDPG and its analogs), inorganic phosphate (intracellular and extracellular), five phospholipid metabolites including PE, phosphocholine (PC), GPE, GPC, and an macromolecular metabolite peak. These metabolites were generally well-dispersed in the acquired spectra at 7-Tesla, allowing accurate quantification based on lineshape fitting analysis of individual ^31^P peaks. Total-ATP (t-ATP) was calculated by averaging the α-, β-, and γ-ATP resonances. The brain ^31^P spectra revealed two distinct peaks for Pi, assigned intra- and extracellular Pi, the latter including contributions from both interstitial and intravascular spaces (Ren et al., 2018). For the membrane phospholipid metabolite index, phosphomonoesters (PMEs) were calculated by summation of PE and PC, whereas phosphodiesters were by summation of GPE and GPC. The metabolite concentrations, BEM index, and membrane phospholipid metabolite index (PMEs/PDES), were measured from the integral of each individual metabolite peaks in the fully relaxed ^31^P spectra, whereas the cellular pH and Mg^2+^ concentration were derived from the chemical shift measurements in the summed spectra at various TRs, which provided high-SNR spectral data. Table 2 summarizes the definitions of all the indices.

### Evaluation of Mg^2+^ and Intracellular pH Concentration in the Parieto-Occipital Regions of the Brain

Free magnesium (Mg^2+^) concentrations in the parieto-occipital regions of the brain were calculated using the chemical shift difference between α- and β-ATP (δ_α–β_ in ppm) with PCr as a reference point (δ _PCr_ = 0) (Ren et al., 2015).

(1) $$\left[Mg^{2+}\right]=k_{d}\frac{\delta_{ATP}-\delta_{\alpha -\beta }}{\delta_{\alpha -\beta }-\delta_{MgATP}}$$

where *k*_*d*_ (dissociation constant of MgATP) = 0.05 mM and the limiting shift constant δ_*ATP*_ = 10.82 ppm and δ_*MgATP*_ = 8.32 ppm.

pH was calculated from the chemical shift of the corresponding Pi (internal) peaks (δ_*Pi*,_ in ppm) in reference to PCr (δ_PCr_ = 0 ppm) (Ren et al., 2015).

(2) $$pH=pKa+log\frac{\delta_{pi}-\delta_{a}}{\delta_{b}-\delta_{pi}}$$

where pKa (deprotonation constant) = 6.73, δ_*a*_ = 3.275 ppm (for acidic protonated species H_2_PO_4_^–^) and δ_*b*_ = 5.685 ppm (for basic protonated species H_2_PO_4_^–^) were used in the data analysis.

### Statistical Analysis

A linear regression model was used to investigate the relationship of BEM indices using PCr/t-ATP (reflective of energy reserve), Pi/t-ATP (reflective of energy consumption), PCr/Pi (reflective of a metabolic state), regulatory co-factors (Mg^2+^ and pH), and the membrane phospholipid index (PMEs/PDEs) in the parieto-occipital brain region with cognitive performance across the domains of EFs, memory, attention, and visuo-spatial skills. Specifically, all the indices and individual metabolite along with intracellular pH were first transformed by using *f*(*x*) = log(*x*−*a*), whereas intracellular Mg^2+^ was transformed using where *f*(*x*) = −log(*b−x*) where “*x*” is the raw score of the specific metabolite for each participant, and “*a*” or “*b*” are metabolite-specific constants. The metabolite-specific constants were used to symmetrize each metabolite distribution and reduce undue leverage of the single participants in the regression. Once the indices, Mg^2+^, and pH values were transformed, the linear regression model was used to test the association with neurocognitive measures across the domains of EFs, memory, attention, and visuo-spatial skills.

## Results

### Brain Phosphorous Metabolites at 7T

**FIGURE 1:**
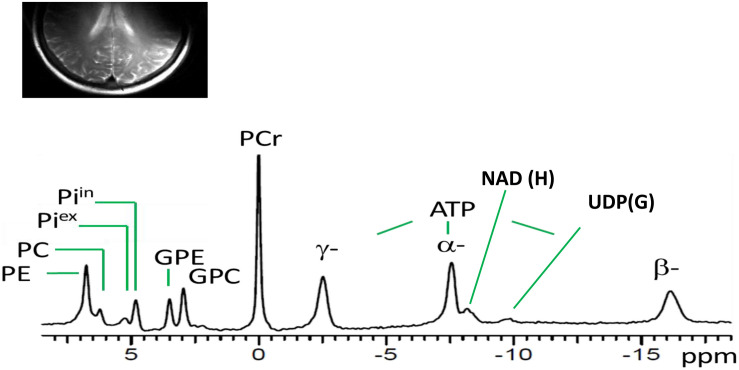
Spectral display of high-energy phosphate and membrane phospholipid phosphorus metabolites from the parieto-occipital lobe of amnestic mild cognitive impairment individual. PE, phosphoethanolamine; PC, phosphocholine; Pi^ex^ and Pi^in^ inorganic phosphate external and internal, respectively; GPE, glycerophosphoethanolamine; GPC, glycerophosphocholine; PCr, phosphocreatine; ATP forms: α, β, and δ adenosine triphosphate; NAD, nicotinamide adenine dinucleotide; UDPG, uridine diphosphate glucose.

Figure 1 shows a typical ^31^P MR spectrum acquired from the human brain parieto-occipital lobes using a partial volume-coil at 7T. A total of 13 metabolites are clearly identifiable.

### Relationship of BEM and Membrane Phosphate Metabolite Indices in the Parieto-Occipital Lobes With Cognitive Performance in aMCI

We investigated the relationships across *BEM indices* measured using PCr/t-ATP (reflective of energy reserve), Pi/t-ATP (reflective of energy consumption: extracellular-Pi/t-ATP and intracellular-Pi/t-ATP), and PCr/Pi (reflective of a metabolic state) and its regulatory co-factors (intracellular Mg^2+^ and pH) followed by *membrane phospholipid metabolite index* (PMEs/PDEs) in the parieto-occipital lobes with cognitive performance of EF (complex abstraction, innovation, inhibition and switching, conceptual reasoning, and working memory), memory (episodic), attention, and visuo-spatial skills. Age and education had no appreciable effects on the high-energy phosphate and membrane phospholipid indices, nor on the cognitive measures in our sample (Supplementary Table S1). Therefore, we did not adjust for age and education in our final analyses of BEM indices and cognitive performance.

#### BEM Index: Cognitive Correlation

In the BEM index and neurocognitive relationship, we found four significant results. First, the extracellular-Pi/t-ATP ratio was inversely related with performance of attention [selective auditory attention-trail 2: (*b* = −0.0018, *t* = −2.254, *p* = 0.038)]; whereas intracellular-Pi/t-ATP ratio was trending to be inversely proportional with the performance on the EF domain of inhibition and switching [Trails B: (*b* = −13.867, *t* = −2.029, *p* = 0.0585)]. Second, the energy reserve indexed by PCr/t-ATP was inversely related with performance on immediate recall of cognitive memory screen [CVLT: (*b* = −0.0138, *t* = −3.272, *p* = 0.004)]. Finally, the regulatory co-factors of BEM index intracellular pH was also inversely associated with attention [selective auditory attention-trail 2: (*b* = −285.95, *t* = −2.172, *p* = 0.044)], whereas Mg^2+^ was positively associated with visual-spatial skills [Trails A: (*b* = −12.471, *t* = 2.219, *p* = 0.040)].

In view of a previous study by Rijpma et al. (2018) in AD for observation of a reduced level of PCr/Pi, an indicator of metabolic state, we also investigated the relationship of PCr/Pi ratio with cognition. A significant inverse relationship was found between the PCr/Pi index and performances on EF of innovation [TOSL: (*b* = −0.78, *t* = −2.18, *p* = 0.044)], and delayed recall of cognitive memory screen [CVLT: (*b* = −2.43, *t* = −2.278, *p* = 0.007)].

**FIGURE 2:**
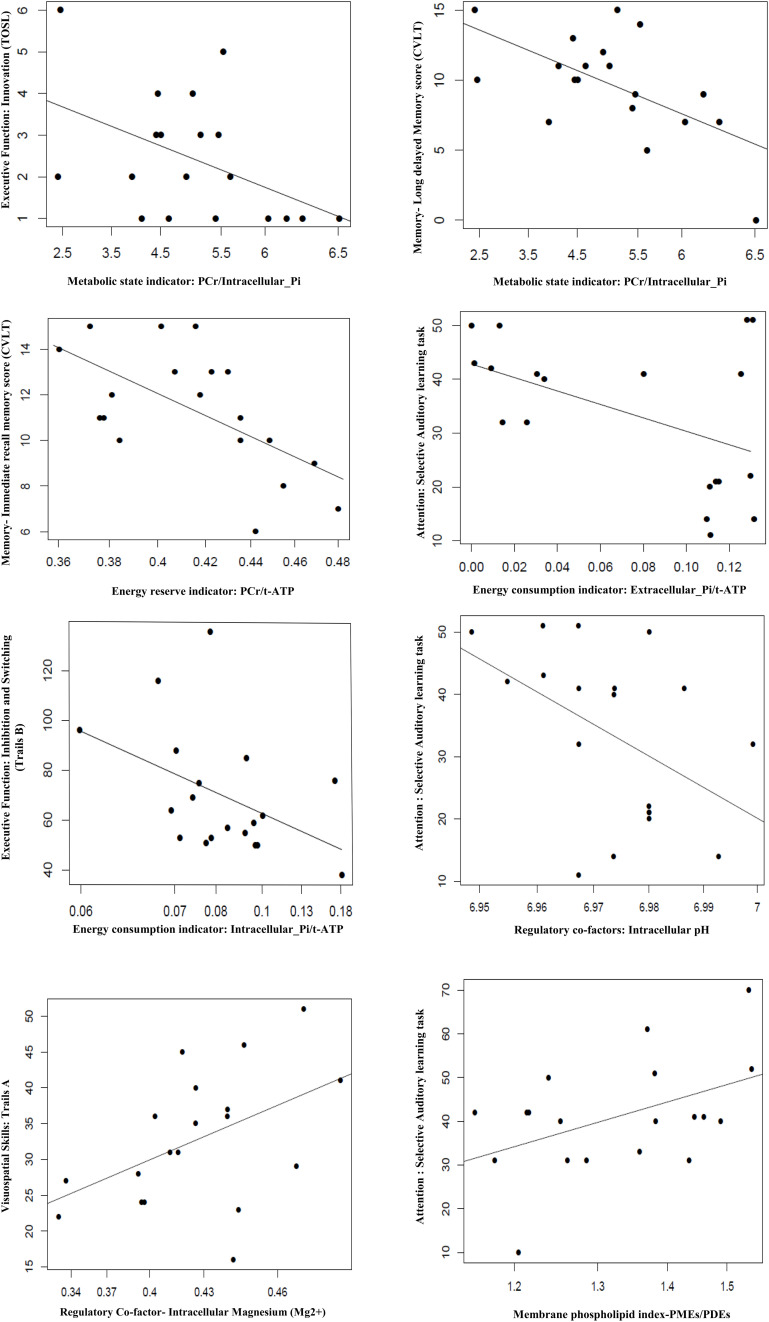
Association of BEM, membrane phospholipid indices, and regulator co-factors in the parieto-occipital brain region with cognitive performance in amnestic mild cognitive impairment (*p* ≤ 0.05). TOSL, Test of Strategic Learning; CVLT, California Verbal Learning test; intracellular_Pi, inorganic phosphate (intracellular); extracellular_Pi, inorganic phosphate (extracellular); PCr, phosphocreatine; t-ATP (total adenosine triphosphate: sum of α-ATP, β-ATP, and γ-ATP); PMEs, phosphomonoesters; PDEs, phosphodiesters.

See Figure 2 and Table 5 for details.

**TABLE 5:** Linear regression model: association of BEM and membrane phospholipid indices in the parieto-occipital brain areas with neurocognitive and screening cognitive measures in mild cognitive impairment.

Metabolite: Neurocognitive relationship	Results		
	*b* (SE)	t-Statistics (df = 17)	*p*-Value
**BEM indices and neurocognitive relationship**			
PCr/t-ATP: Immediate recall on screening memory measure (CVLT)	–0.0138(8.556)	–3.272	0.004*
Extracellular_Pi/t-ATP: Attention (selective auditory task-Trail 2)	−0.0018(55.385)	–2.254	0.038*
Intracellular_Pi/t-ATP: Executive function (switching and inhibition: Trails B)	−13.867(6.836)	–2.029	0.0585
PCr/intracellular_Pi (oxidative phosphorylation rate): Executive function (innovation: TOSL)	−0.78(0.36)	–2.18	0.044*
PCr/intracellular_Pil: Delayed recall on screening memory measure (CVLT)	−2.43(0.79)	–2.78	0.007*
pH: Attention (selective auditory task-Trail 2)	−285.95(131.66)	–2.172	0.044*
Mg^2+^: Attention (Trails A)	12.471 (5.620)	2.219	0.040*
**Membrane phospholipid metabolite index and neurocognitive relationship**			
PMEs/PDEs: Attention (selective auditory task-Trail 1)	5.92 (2.77)	2.14	0.047*

*DKEFS, Delis–Kaplan Executive Function System™; TOSL, Test of Strategic Learning; CVLT, California Verbal Learning Test. p-values < 0.05^∗^ (significant).*

#### Membrane Phospholipid Metabolite Index: Cognitive Correlation

The membrane phospholipid metabolite index (PMEs/PDEs) was positively associated with cognitive domain of attention [selective auditory attention-Trail 1: (*b* = 5.92, *t* = 2.14, *p* = 0.047)].

See Figure 2 and Table 5 for details.

## Discussion

The present exploratory pilot study investigated two main goals in aMCI, a heterogeneous and unstable clinical population. This group is at higher risk of developing AD/dementia than the cognitively normal population, although many with aMCI may not progress. Our main goal was to establish a methodology and test the feasibility to measure BEM related high-energy phosphate and membrane phospholipid metabolites precisely at UHF magnetic strength 7-Tesla using a partial volume-coil ^31^P MRS technique in the parieto-occipital region of the brain in a population with variability as defined by its nature. Our results showed that we were able to resolve 13 of ^31^P peaks with well-separated spectral resolution of high-energy phosphate and membrane phospholipid metabolites. The enhanced spectral resolution suggests this methodology may provide a feasible way to further explore BEM in more extensive comparative studies with healthy controls and those affected with AD in the future at UHF magnetic strength. Next, we investigated if the BEM indices derived from measuring the high-energy phosphates and membrane phospholipid metabolites at 7T showed a correspondence with cognitive performance (BEM-Cognitive correlates). Overall, we hypothesized an inverse relationship across BEM indices—energy reserve, energy consumption, and metabolic state—along with regulatory co-factors pH and Mg^2+^ and membrane phospholipid index with cognitive performance supported by previous work from Ashraf et al. (2015), Croteau et al. (2018), and Corriveau-Lecavalier et al. (2019). Our results support our assumptions of an inverse relationship in aMCI with the exception of Mg^2+^ and the membrane phospholipid index, which were positively associated with visuospatial domain and attention, respectively.

### Population of Interest

An increasing body of research is focused on investigating pathophysiologic changes in the *in vivo* human brain with the goal to track and diagnose the onset of dementia as early as possible, particularly in populations at higher risk, such as aMCI. Although this cohort has an elevated risk for developing Alzheimer’s, the evidence that many will not develop dementia may make this an informative population to study, as a preliminary step. Due to variability of the disease progression, identification of early biological markers is an important goal in developing effective therapeutics. In prior research, seeking to better understand BEM, the research to date focused predominantly on either healthy controls or disease populations like AD at lower magnetic strength, with a knowledge gap regarding the potential role of BEM on cognition in aMCI. The current work expands on these efforts by reporting on the first, albeit small, pilot study in aMCI to explore the feasibility of acquiring precise data at UHF magnetic strength 7T using ^31^P MRS to study BEM-cognitive correlations.

### Methodological Approach to Measure BEM: ^31^P MRS at 7T in aMCI

Our first goal of this study was to identify a distinct spectral display of high-energy phosphate and membrane phospholipid metabolites using ^31^P MRS at 7T. The results revealed it was possible to resolve and identify a high-quality spectral display with improved SNR of the high-energy phosphate and membrane phosphorus metabolites in the parieto-occipital lobes using the UHF MRI. What is interesting to note is that the spectral resolution of brain phosphorus metabolites in this study was similar to previous studies in healthy young adult brains and muscle (Ren et al., 2015) and heart (Rodgers et al., 2014) at 7T. Moreover, the use of 7T allowed clear separation of multiple phosphorus metabolites with similar chemical shifts in the spectra collected from the parieto-occipital region. With improved SNR in the summed spectra, we were able to fully resolve PMEs into PE and PC, PDEs into GPC and GPE, and Pi into intra- and extracellular compartments, along with observation of NAD(H) and UDP(G). At UHF MRI, the α- and β-ATP signal appeared to be an easy-to-fit single peak on the spectral display due to improved SNR of the phosphorus metabolites. It is worthy to note that the α-ATP chemical shift can be affected by the NAD(H) signal, a shift that cannot be resolved at a lower magnetic field. Thereby, the easy-to-fit singlet of α- and β-ATP allowed us to calculate the concentration of free Mg^2+^, an important regulatory co-factor of BEM in the *in vivo* human brain. Overall, the ^31^P MRS data acquisition methodology with improved SNR provided indirect help in accurately measuring the regulatory co-factors (intracellular Mg^2+^ and pH), which are essential metabolic sensors to provide neuronal environmental cues alterations in health and disease progression (Rabhi et al., 2017). This capability was previously limited due to the use and insensitivity of lower magnetic strengths such as 1.5-Tesla (Smith et al., 1995; Lotti et al., 1996; Forlenza et al., 2005) or 3-Tesla (Mandal et al., 2012; Rijpma et al., 2018).

Another methodological contribution of this study was the resolution of Pi into two separate peaks, intracellular and extracellular compartments (Ren et al., 2018). The extracellular Pi pool includes the signal of Pi from both the vascular and interstitial space plus the space occupied by cerebrospinal fluid (CSF). One hypothesis that could be explored in subsequent studies is whether brain atrophy leads to increased extracellular space and reduced intracellular space, assuming invariant extracellular-Pi is a crucial biomarker as the disease emerges and progresses. Prior work in MCI and AD supported the postulation that low level of serum or blood phosphorus, that is, phosphorus levels, was significantly associated with higher cerebral amyloid deposition when compared to healthy controls matched for age, sex, and apolipoprotein ε4 genotype and memory scores of MMSE (Park et al., 2017). Park et al. (2017) findings motivated us to explore a relationship between phosphorus metabolites in the brain with cognition in MCI. Building on this prior evidence, the current work expands the knowledge by going one step further in measuring phosphorus metabolites, an indicator of BEM directly using ^31^P MRS at 7T, followed by exploring the relationship of these phosphorus metabolites with cognition in aMCI.

To date, research in MCI has focused largely on the macroscopic pathological contributing factors associated with neurodegeneration, such as the accumulation of amyloid and structural alteration (Mufson et al., 2012; Oeltzschner et al., 2019). However, there is a desire to better understand the microscopic biological mechanisms taking place inside the cells of the *in vivo* human brain, now that researchers have access to more advanced imaging technology and analytics. As protocols develop and are tested to measure intracellular mechanisms, it may improve the predictive capabilities of which profiles progress toward the disease compared to non-converters. Our preliminary work using ^31^P MRS at 7T opens the door to employ this new methodology to advance our understanding of the biological mechanisms like BEM, that is, bioenergetics and membrane phospholipid metabolism occurring inside the brain cells in aMCI, a heterogeneous cohort.

### BEM-Cognitive Correlates

Our second goal of the study was to investigate the association of BEM and membrane phospholipid metabolite indices in the parieto-occipital brain regions, with the cognitive performance of EFs, attention, memory, and visuospatial skills. We measured BEM indices using PCr/t-ATP (reflective of energy reserve), Pi/t-ATP (reflective of energy consumption: extracellular-Pi/t-ATP and intracellular-Pi/t-ATP), and PCr/Pi (reflective of a metabolic state), along with co-regulatory factors intracellular pH and Mg^2+^ concentrations in the parieto-occipital lobes (see Table 2 for details). The membrane phospholipid index ratio of PMEs (the significant precursors of phospholipids contributing to membrane synthesis) to PDEs (significant phospholipid products breakdown) indicated neuronal and non-neuronal membrane integrity (Ross and Sachdev, 2004) measured in the parieto-occipital lobes. An inverse relationship was observed with BEM indices and cognitive performance except for Mg^2+^ and membrane phospholipid index, which were significantly associated with visuospatial domain and attention, respectively.

The results of the inverse relationship of BEM indices and cognition support that individuals with higher BEM in the parieto-occipital lobes performed lower on cognitive domains of memory and EF. We offer one plausible hypothesis that subsequent research could explore is whether these low cognitive performers with higher BEM indices may represent individuals at greater risk of progressing toward AD in longitudinal work. This possibility is based on prior work on brain metabolism using ^18^FDG-PET (Ashraf et al., 2015; Croteau et al., 2018). The present work complements prior findings showing glucose hypermetabolism in MCI when compared to age-matched healthy controls, in parietal (Croteau et al., 2018) and occipital lobes (Ashraf et al., 2015). Although these two studies focused on different brain regions, they both offered a similar explanation for their findings; that is, the glucose hypermetabolism in parietal and occipital lobes represented a compensatory mechanism in MCI compared to healthy controls.

A compensatory mechanism can be interpreted as a process whereby the brain is adapting to neural changes attributed to a variety of factors, such as brain aging or neurodegenerative processes (Bobkova and Vorobyov, 2015). This adaptive mechanism was explained as “neuroplasticity” in Ashraf’s study. It is important to note that, at some point, the compensatory phenomenon of the brain’s systems, such as higher BEM observed in our study, may not have been adequate to support the same level of cognitive performance as cognitively normal individuals (Croteau et al., 2018). To date, impairments across EF, memory, and attention are thought to rely on the anterior brain networks of the frontal, temporal, and anterior part of the parietal lobes (Kim et al., 2019) with limited understanding of investigating early neurometabolic changes in the posterior part of the brain-parietal and occipital lobes. Thus, the current work lays a foundation to examine the correlates of BEM in posterior part of the brain, that is, parieto-occipital regions of the brain with cognition in aMCI, a group where some are at risk of developing dementia while others are stable either in the condition or revert to normal.

In contrast to the inverse relationship of BEM indices with cognitive performance, two independent variables (regulatory co-factor: Mg^2+^ and membrane phospholipid index) also showed significant association with specific cognitive domains but in different directions in aMCI. One was a higher Mg^2+^ concentration and associated with the higher performance on the visuospatial task, and other was a positive association of membrane phospholipid index (PMEs/PDEs) with the performance on the attention task. One possible explanation of the deviation of the inverse relationship of Mg^2+^ in the parieto-occipital lobes with cognition is that the posterior parietal and occipital brain regions are part of the dorsal stream of the visual system that aids in recognizing objects in space with detection and analyzing the movements (Hebart and Hesselmann, 2012). Thereby, a visuospatial task, which is primarily controlled by the parietal and occipital lobes, could depend on higher energy requirements in the form of higher Mg^2+^ level, an essential co-factor for ATP synthesis. Whereas the other significant positive correlation of the membrane phospholipid index with the cognitive domain of attention in the region of interest may again support the theory of neuroplasticity/compensatory mechanism as proposed by Ashraf’s et al. (2015) in MCI. We speculate that neuroplasticity of the parietal and occipital lobe may have higher turnover of membrane phospholipid index to support failing anterior lobe network. In general, though this study generates a lot of hypothesis that needs to be tested in future studies, still it adds to the existing knowledge that BEM and membrane phospholipid indices are associated with cognition in a cohort with mixed progression and reversion rate.

In sum, this study represents an early step to elucidate the methodologies of BEM and membrane integrity using partial volume-coil ^31^P MRS at UHF magnetic strength 7T in aMCI. We were able to show the feasibility of measuring the high-phosphate energy and membrane phospholipid metabolites with higher sensitivity and spatial resolution when compared to the strength of lower magnetic fields, such as 1.5T and 3T, in a heterogeneous population. This pattern of findings supports the viability of ^31^P MRS at 7T to explore further the brain’s energy requirements and its association with cognition to motivate additional research. We propose that future research using volume-coil ^31^P MRS at 7T could enhance our understanding of the role of mitochondrial contributions, which play an essential role in BEM linked to brain pathology in at risk populations in whom the disease may or may not progress from healthy aging to AD. Moreover, the potential of such an approach is strengthened by the finding of a relationship between BEM and membrane phospholipid indices in parieto-occipital lobes with cognitive performance across the domain of EF, memory, attention, and visuospatial skills in cognitively compromised brain of aMCI.

### Limitations

We interpret the findings of this study in aMCI cautiously due to its limitations. First, the study sample size was small and lacked a cognitively normal age-matched control group or AD cohort for comparison. However, even with our small sample size and lack of a control group, we were able to show that BEM and membrane phospholipid indices, an indicator of neurometabolic changes in the parieto-occipital brain areas, are associated with performance of cognition especially in the area of memory, EF, and attention in aMCI significantly. This study focused on exploring a relationship of BEM-cognitive correlates at a single point of time. Future research is needed to follow individuals with aMCI longitudinally to see if early markers could be identified to better predict who is at risk for developing AD/dementia and who is less likely to progress. Second, the use of partial volume-coil ^31^P MRS limited our ability to support the theory of the compensatory mechanism in the early stages as the BEM indices of the anterior part of the brain, that is, frontal and temporal were not measured. Nonetheless, the use of the partial volume-coil in this research helped us to understand the relationship of a BEM and cognition in aMCI, specifically in brain regions that have been shown to probable predictor sites in the brain in subset of MCI individuals that progress toward AD using ^18^FDG-PET (Ashraf et al., 2015; Croteau et al., 2018).

## Conclusion

Our findings show a highly resolved spectral display of high-energy phosphate and membrane phospholipid metabolites using ^31^P MRS at 7Tesla in aMCI. We also observed an inverse relationship between higher BEM indices with the lower performance of EFs, memory, and attention. The salient contribution of this pilot study is that it adds to the growing body of evidence that BEM can be measured accurately and appears to be associated with cognitive performance in this heterogeneous cohort. It also lays the foundation for future efforts to investigate if BEM mechanisms synergistically with amyloid and tau proteins, which are the hallmarks of the disease as potentially understand upstream triggers that aggravate the pathophysiological mechanism of the disease. Finally, longitudinal follow-up of an aMCI population would be of interest to test predictive ability of BEM, deploying imaging technology at 7 Tesla, as to which individuals will develop dementia and which will not, based on changes at the molecular level. This novel approach offers a novel research approach to advance our understanding of early biomarkers of those at risk for dementia to complement current endeavors.

## Data Availability Statement

The raw data supporting the conclusions of this article will be made available by the authors, without undue reservation.

## Ethics Statement

The studies involving human participants were reviewed and approved by IRB, The University of Texas Southwestern Medical Center and The University of Texas at Dallas. The patients/participants provided their written informed consent to participate in this study.

## Author Contributions

ND has been involved in the analysis and writing of the whole manuscript. JR has been involved in instrumental in the analysis of MRS data along with ND. JS, biostatistician was involved in the analysis, whereas AR helped in the recruitment of MCI participants. SC was the principal investigator of the study and mentored in the writing of the manuscript. All authors contributed to the article and approved the submitted version.

## Conflict of Interest

The authors declare that the research was conducted in the absence of any commercial or financial relationships that could be construed as a potential conflict of interest.
