# Supplementary material for: Relationship of Parieto-Occipital Brain Energy Phosphate Metabolism and Cognition Using 31P MRS at 7-Tesla in Amnestic Mild Cognitive Impairment
Source: Front Aging Neurosci. 2020 Aug 28;12:222. doi: 10.3389/fnagi.2020.00222 (PMC7483543; doi:10.3389/fnagi.2020.00222)
Supplement: Supplementary file 1 [file Table_1.DOCX]

**Table 1 - Linear regression model of BEM and membrane phospholipid indices and cognitive performance measure on age and education respectively.**

| **AGE-adjusted scores** |  |  |  |  |
| --- | --- | --- | --- | --- |
|  | B | se(B) | t-stat | p-val |
| PCr/t-ATP | 0.00214 | 0.00192 | 1.111 | 0.282 |
| Intracellular_Pi/t-ATP | 0.00169 | 0.02686 | 0.063 | 0.950 |
| PCr/Pi_intracellular | 0.01093 | 0.01225 | 0.892 | 0.385 |
| Magnesium (Mg^2+^) | 0.00162 | 0.01193 | 0.136 | 0.894 |
| pH | -0.00011 | 0.00076 | -0.143 | 0.888 |
| PMEs/PDEs | -0.01324 | 0.00642 | -2.064 | 0.055 |
|  |  |  |  |  |
|  | B | se(B) | t-stat | p-val |
| Attention: Strategic Attention task | -0.55001 | 0.44856 | -1.226 | 0.237 |
| Executive function: TOSL | -0.03086 | 0.05039 | -0.612 | 0.548 |
| Visuospatial-skills: Trails A | 0.09367 | 0.31318 | 0.299 | 0.769 |
| Memory: DSFS | -0.00469 | 0.06497 | -0.072 | 0.943 |
| Memory-Delayed Recall : CVLT | -0.07458 | 0.12199 | -0.611 | 0.549 |
| Memory-Immediate Recall :CVLT | -0.06229 | 0.35570 | -0.175 | 0.863 |

| **EDUCATION-adjusted scores** |  |  |  |  |
| --- | --- | --- | --- | --- |
|  |  |  |  |  |
|  | B | se(B) | t-stat | p-val |
| PCr/t-ATP | -0.0245 | 0.0485 | -0.505 | 0.620 |
| Intracellular_Pi/t-ATP | 0.8465 | 0.6252 | 1.354 | 0.194 |
| PCr/Pi_intracellular | -0.4504 | 0.2870 | -1.569 | 0.135 |
| Magnesium (Mg^2+^) | 0.0011 | 0.2923 | 0.004 | 0.997 |
| pH | -0.0141 | 0.0184 | -0.770 | 0.452 |
| PMEs/PDEs | -0.0900 | 0.1744 | -0.516 | 0.613 |
|  |  |  |  |  |
|  | B | se(B) | t-stat | p-val |
| Attention: Strategic Attention task | 9.6216 | 11.2234 | 0.857 | 0.403 |
| Executive function: TOSL | -0.7880 | 1.2332 | -0.639 | 0.531 |
| Visuospatial-skills: Trails A | -1.4722 | 7.6835 | -0.192 | 0.850 |
| Memory: DSFS | 0.1236 | 1.5914 | 0.078 | 0.939 |
| Memory-Delayed Recall : CVLT | 1.9574 | 2.9833 | 0.656 | 0.521 |
| Memory-Immediate Recall : CVLT | 2.4935 | 8.6999 | 0.287 | 0.778 |

Abbreviations: TOSL: Test of strategic learning, CVLT: California Verbal Learning test, intracellular_Pi: Inorganic phosphate (intracellular), extracellular_Pi: Inorganic phosphate (extracellular), PCr: Phosphocreatine, t-ATP (total adenosine triphosphate: sum of α-ATP, β-ATP, and γ-ATP), PMEs: phosphomonoesters, PDEs: phosphodiesters.

Figure 1: B1 calibration: Phosphocreatine (PCr) color map overlaying with a sagittal T2w MR image, showing the contribution of PCr signals dominantly from brain parieto-occipital region (> 95%). Color map reconstructed from ^31^P MRSI data acquired with a 12 x 8 (FH x AP) matrix at 7-Tesla.


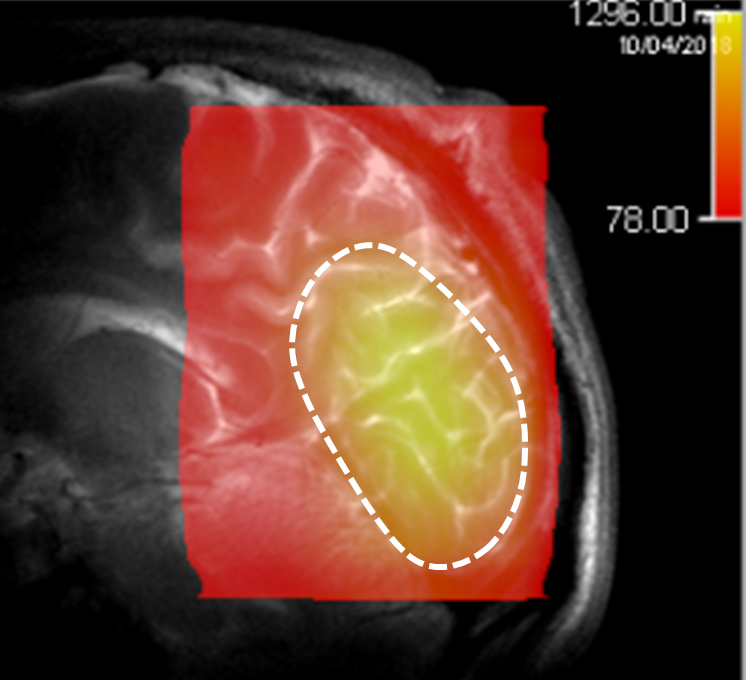


PCr Map

Abbreviations: PCr-phosphocreatine
